# A COVID-19 Infection Prediction Model in Egypt Based on Deep Learning Using Population Mobility Reports

**DOI:** 10.1007/s44196-023-00272-z

**Published:** 2023-05-30

**Authors:** Nour Eldeen Khalifa, Ahmed A. Mawgoud, Amr Abu-Talleb, Mohamed Hamed N. Taha, Yu-Dong Zhang

**Affiliations:** 1 Information Technology Department, Faculty of Computers and Artificial Intelligence, Cairo University, Giza, Egypt; 2 Computer Science Department, Faculty of Computers Science, University of People, Pasadena, USA; 3 School of Computing and Mathematic Sciences, University of Leicester, East Midlands, LE1 7RH UK

**Keywords:** Deep learning, COVID-19, Prediction model, Regression model, Egypt

## Abstract

The rapidly spreading COVID-19 disease had already infected more than 190 countries. As a result of this scenario, nations everywhere monitored confirmed cases of infection, cures, and fatalities and made predictions about what the future would hold. In the event of a pandemic, governments had set limit rules for the spread of the virus and save lives. Multiple computer methods existed for forecasting epidemic time series. Deep learning was one of the most promising methods for time-series prediction. In this research, we propose a model for predicting the spread of COVID-19 in Egypt based on deep learning sequence-to-sequence regression, which makes use of data on the population mobility reports. The presented model utilized a new combined dataset from two different sources. The first source is Google population mobility reports, and the second source is the number of infected cases reported daily “world in data” website. The suggested model could predict new cases of COVID-19 infection within 3–7 days with the least amount of prediction error. The proposed model achieved 96.69% accuracy for 3 days of prediction. This study is noteworthy since it is one of the first trials to estimate the daily influx of new COVID-19 infections using population mobility data instead of daily infection rates.

## Introduction

Since its discovery in December 2019, coronavirus (COVID-19) has spread throughout the world [1]. The spread of COVID-19 is accelerated when people can relate to each other. To keep the virus from spreading, travel limitations and frequent hand washing are necessary. The most frequent symptoms of this illness are fever and dyspnea [2]. Other signs, such as chest pain, sputum production, and hoarseness, may also occur. The cause of pneumonia, which has a 5.8% fatality rate, can develop from COVID-19 [3]. The number of deaths from COVID-19 is 5% higher than the number of deaths from the 1918 Spanish flu outbreak.

**Fig. 1:**
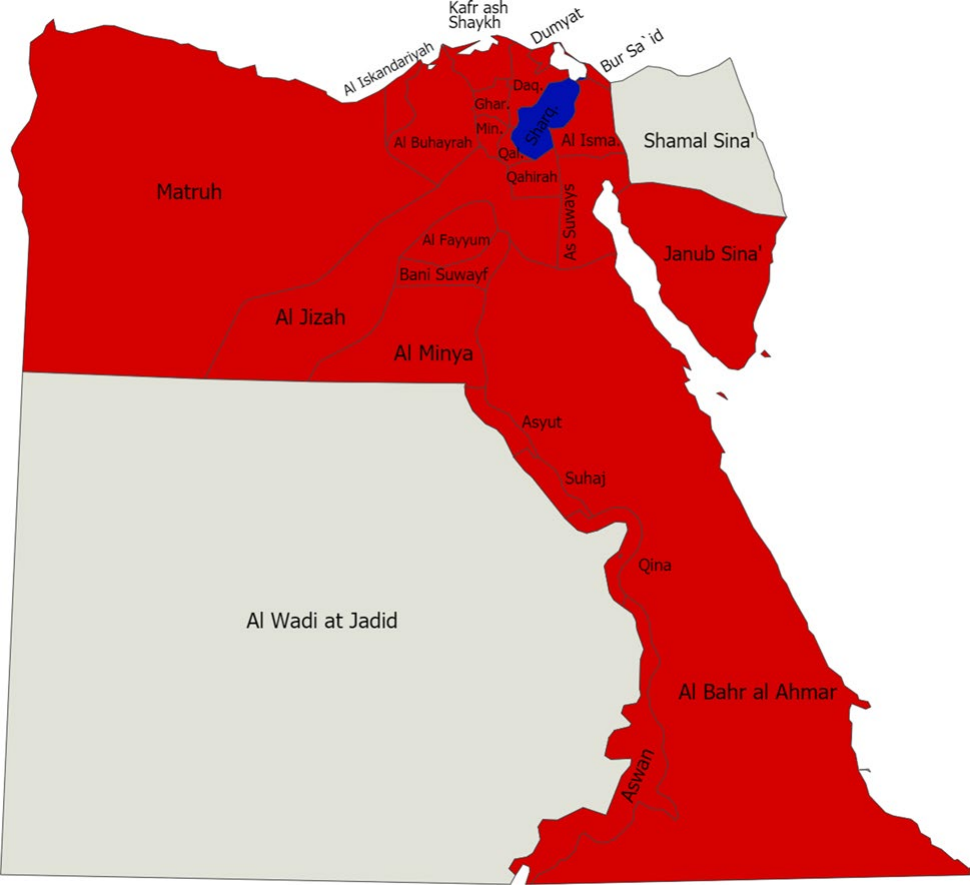
On May 13, 2020, the map will show the local government areas in Egypt where COVID-19 has confirmed infection cases (red) or where infection cases are suspected (blue) [43]

Medical researchers from many fields are working hard to find ways to fix the problems with the virus and stop it from spreading [4]. As shown in Fig. 1, on September 6, 2020, Egypt had the second-highest count of COVID-19 cases in Africa. Luxor, a popular tourist destination in southern Egypt, is the likely source of the epidemic, according to the Egyptian Health Ministry. A maximum of 5,511 deaths and 99,712 reported cases of coronavirus were confirmed as of September 6, 2020. Fresh cases and deaths, as shown by the Egyptian Ministry of Health’s latest data, have increased significantly lately, raising fears of another new wave [5].

The Egyptian government has formulated and developed plans to regulate and limit the pandemic’s effects on both human health and business continuity. Major economic, manufacturing, and logistic activities were halted mostly from February 15th, 2020, until January 14th, 2022 (overall 700 records). Furthermore, several sectors operated at low efficiency to reduce the amount of time employees spent together [6]. Corporations that were obligated to offer necessities and services were exempt. Emergency declarations were made at various ports owing to COVID-19. Furthermore, medical facilities were given the task of caring for infected patients. To enhance public perception of the worldwide epidemic problem, the government initiated several projects. Since institutions in low- and middle-income nations cannot keep up with epidemics, raising awareness among the general public is a critical component in limiting the spread of infectious diseases. Moreover, free vaccinations are being administered to all people, with a focus on healthcare experts and those with serious illnesses [7].

**Fig. 2:**
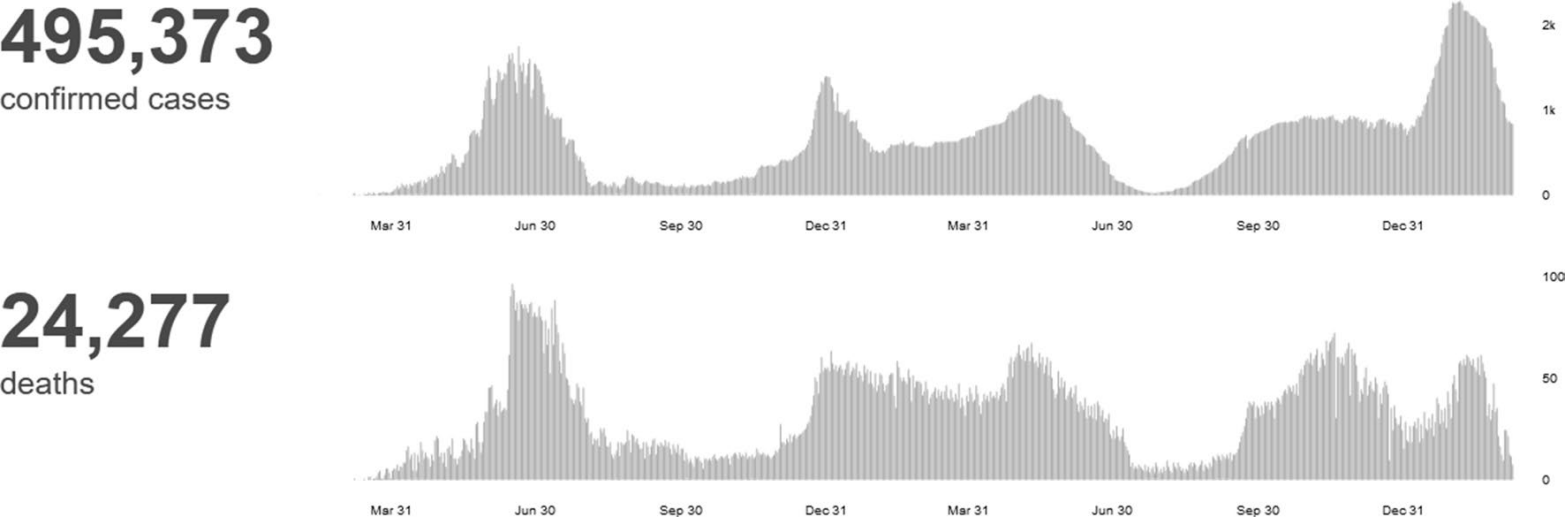
In Egypt, from 3 January 2020 to 7:33 pm CET, 18 March 2022, there have been 495,373 confirmed cases of COVID-19 with 24,277 deaths. (World Health Organization ‘WHO’, March 2022)

Up until March 21, 2022, a total of 76,977,234 doses of vaccine were given to 31.11 million people, which is 30.5% of the population. Figure 2 shows the number of death cases in Egypt from the 3rd of January 2020 till the 21st of March 2022 [8].

Observing a certain event through time and projecting its future response can be termed time-series analysis. Numerous algorithms can then be used to predict the time-series data. There are three types of these designs. Methods are based on statistics, machine learning, and deep learning. Each has its strengths and weaknesses. Statistical methods may not be appropriate for addressing complicated and non-linear data patterns. On the other hand, machine-learning techniques possess the capability to manage more complex data patterns and can assemble insights from historical data to formulate predictions. However, they may require a substantial volume of data for training and may not be able to understand long-term dependencies in the data. Deep learning methods can handle large amounts of data and capture long-term dependencies but require large computational resources and are difficult to explain.

### Problem Definition and Study Contributions

The problem definition for this study is to address the global pandemic of COVID-19 and to predict the spread of the disease in Egypt. It is a means of informing public health policy and decision-making in many sectors such as the tourism sector and health sector. The objective is to develop a model that can predict new cases of COVID-19 infection with high accuracy and low prediction error, to help prevent the spread of the disease and save lives.

The primary contribution of this study is the ability to forecast the number of infected cases in Egypt for the short term (3 days or 1 week) by utilizing deep learning on population mobility reports instead of the number of infection reports. The following is a condensed summary of the most significant findings from the study: 

- A new dataset from two different sources. The first source is Google population mobility reports, and the second source is the number of infected cases reported daily “world in data” site.
- A deep learning model using multivariate sequence-to-sequence regression based on population mobility reports.
- Predicting the number of Egyptians who would be infected with the highest accuracy possible with 96.69% for 3 days of prediction.

### Paper Organization

In Sect. 2, a literature review of several recent related research was investigated. Section 3 presents the dataset characteristics. Section 4 illustrates the proposed model phases and design. Section 5 is the main discussion of the output results along with their efficiency and limitations. Section 6 presents the conclusion that summarizes the overall work in this study with the future directions.

## Literature Reviews

As the COVID-19 pandemic harshly spreads across the world, researchers have conducted numerous studies aimed at comprehending and identifying this disease. A collaborative effort has been made to predict the number of confirmed cases, recoveries, and fatalities [9]. Given the fact that those infected may not exhibit symptoms promptly, it is essential to develop a model or a system for continuously measuring the number of potentially affected individuals. This information is essential to put into effect countermeasures that will effectively curb the virus’s spread.

Many researches were done to monitor and compact COVID-19 such as tracking COVID-19 geographical infections using real-time tweets [10], investigating the role of emerging technology in the fight against the COVID-19 pandemic [11], determining the COVID-19 influence on the electrical industry [12], using machine learning and deep learning models to classify potential coronavirus treatments on a single human cell [13] and more. Numerous types of research focus on the classification and categorization of COVID-19 CT and X-ray images [14]–[17]. Predicting the development of COVID-19 disease and examining its epidemiologic characteristics are important issues that should be thoroughly investigated to reduce outbreak frequency, control output, and allocate medical resources [18].

An approach based on statistics known as the auto-regressive integrated moving average ARIMA is utilized in forecasting. ARIMA has been suggested by numerous researchers to predict the epidemic behavior of diseases including influenza, SARS, and HIV [19]. Table 1 represents a comparative geographical analysis of earlier COVID-19 prediction approaches based on the number of daily infected cases.

**Table 1:** A comparative analysis of the previous COVID-19 forecasting methods based on the number of daily infected cases

References	Countries	Short description	Methods
[20]	India	Forecasting recovery and death of COVID-19 cases	ARIMA approach
[21]	South Africa	COVID-19 infection rate	R statistical technique
[22]	Brazil	Prediction of COVID-19 cases comparative analysis study	Random decision forests—SVR—ARIMA
[23]	Italy, Spain, and France	Monitoring and forecasting COVID-19 infection distribution	ARIMA time series
[24]	Saudi Arabia	Confirmed, retrieved, and fatal COVID-19 cases rate	LSTM model
[25]	Egypt	Predict COVID-19 prevalence	LSTM and gated recurrent unit (GRU) model

The number of known and recovered COVID-19 cases and deaths in India [20] was predicted using an ARIMA technique. Because of the scarcity of medical resources and the restrictions placed on social gatherings, the authors expressed concern about the outbreak spreading rapidly. In South Africa [21], the number of predicted cases was higher than in Nigeria. South Africa has a far higher number of infected people than Nigeria, and there are far more tests conducted in South Africa than in Nigeria. After a 2-month lockout, ARIMA seasonal forecasting software using R statistical technique was used to estimate the number of new and recovered cases in Italy. It was suggested that the quarantine period be extended and that all transport between cities is prohibited.

In Brazil’s infection investigation [22], ARIMA was compared to five different statistical methods to estimate the frequency of COVID-19 in Brazil. The stacking-ensemble learning method, regression models, cubist regression, and SVR are some of the models that fall into this category. In terms of prediction performance, the random decision forests technique, regression models, cubist regression, ARIMA, stacking-ensemble instructional strategy, and support vector regression are ranked from worst to best.

In Italy, Spain, and France [23], the authors have studied that health agencies must anticipate the rate of diabetes to increase monitoring and reallocate resources. Forecasting models help analyze outbreaks and anticipate diseases. This study used ARIMA time-series models to evaluate COVID-19 distribution in Italy, Spain, and France. The study’s conclusions can assist politicians and health officials to plan and provide staff, and critical healthcare centers to handle the situation in these nations in the next weeks or months. Actual data updates improve comparisons and future works.

In Saudi Arabia [24], the authors have proposed a deep learning model that was used to find out how many COVID-19 cases in Saudi Arabia were confirmed, retrieved, and fatal. Official data trained the network. To maximize prediction performance, the ideal hidden layer units and early developmental percentage were decided. The suggested model forecasted 1 week much more accurately than NARANN and ARIMA. The RMSE of predicted results using LSTM is a little less than 11% for ARIMA and 28% for NARANN.

In Egypt [25], the authors used LSTM and gated recurrent unit (GRU) and they applied them to time-series data in three countries: Egypt, Saudi Arabia, and Kuwait. Their results indicate that the LSTM algorithm outperformed the GRU algorithm in predicting death cases in Egypt. The best performance was recorded by an LSTM model with a single layer, with a mean absolute percentage error (MAPE) of 0.44542, a root mean squared error (RMSE) of 29.86051, and a mean absolute error (MAE) of 28.59449. The Egyptian government found these models extremely useful in managing the COVID-19 outbreak over the next few months. Using the concept of drift across multiple data streams [26] can be used to improve different models’ accuracy by detecting the change of error rate. The goal of the study was to investigate and evaluate how well Egypt’s measures to stop the spread of COVID-19 work.

## Dataset Characteristics

The main goal of this research is to predict Egypt’s COVID-19 new infection cases through deep learning using a combined dataset from two sources, the first source is Google mobility reports for Egypt [27] (https://www.google.com/covid19/mobility) and the second source is the number of daily infected cases from the world in data site [28] (https://ourworldindata.org/coronavirus/country/egypt). The following points present the description of those sources in detail:

- Google mobility reports for Egypt: It is the first source of data to be used in our study; a Google Maps-like commodity might be useful in the fight against COVID-19. The purpose of these community mobility findings is to shed light on how regulations designed to tackle COVID-19 have altered the situation [27]. In this research, the columns (pharmacy, parks, transit, workplace, and residential) were extracted from google mobility reports.
- World in data: It is the other source for collecting new daily COVID-19 case data in Egypt. The website launched by Global Change Data Center, which is an English nonprofit organization aims to make knowledge on big problems accessible and understandable [28]. In this research, the number of the daily infected case was extracted from it.

The two sources of data were combined using the date column which relates the mobility reports with the number of daily infected cases. Table 2 presents a sample of data. The combined dataset includes 700 records (each record present for a day) in Egypt and 5 different columns (pharmacy, parks, transit, workplace, and residential) with COVID-19 new infection cases from 15-Feb-2020 until 14-Jan-2022.

**Table 2:** Samples of records for COVID-19 infections cases and mobility reports in five domains (pharmacy, parks, transit, workplace, and residential) from April 22nd to May 1st

Date (2022)	Pharmacy	Parks	Transit	Workplace	Residential	New cases
22 April	− 9.53571	− 32.076	− 36.16	− 25.43	10	169
23 April	− 13.75	− 33.923	− 36.04	− 24.96	10.5	232
24 April	− 11.321	− 50.125	− 60.39	− 26.73	13.24	201
25 April	− 4.7857	− 44.153	− 55.45	− 33.93	14.76	227
26 April	− 1.75	− 38.230	− 47	− 27.63	13.84	215
27 April	− 3.5714	− 41.44	− 49.83	− 25.73	13.96	248
28 April	− 0.5714	− 39.32	− 47.83	− 25.43	12.07	260
29 April	− 0.25	− 37.961	− 47.79	− 24.73	13.76	226
30 April	− 1.3571	− 38.115	− 49.29	− 24.23	14.23	269
1 May	− 2.71428	− 32.666	− 50	− 17.7	10.52	358

The numbers in columns (pharmacy, parks, transit, workplace, and residential) reflect an average of population mobility to those places. For example, on date 22 April 2022, the population mobility to the pharmacy declined by − 9.53571 from the original state baseline of zero. For parks, the population mobility declined by − 32.076 from the original state baseline of zero. For transit, the population mobility declined by − 36.16. For the workplace, the population mobility declined by − 25.43. For residential, the population mobility was increased by 10 (which means people moved to residential areas or stayed in them).

### Data Visualizations

**Fig. 3:**
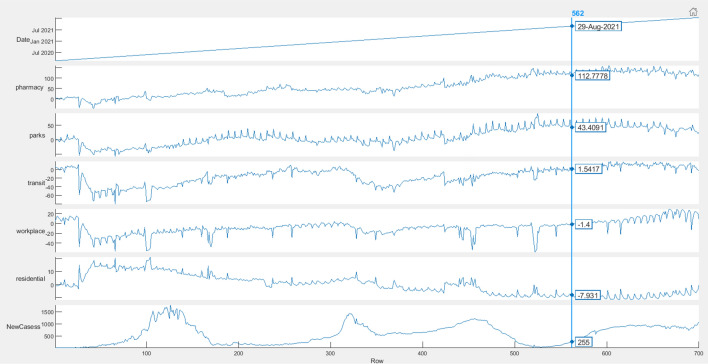
Population mobility reports in five places (pharmacy, parks, transit, workplace, and residential) from 15-Feb-2020 until 14-Jan-2022 with COVID-19 new cases

In order to investigate the quality of the data and discover errors if existed. Data visualization is a necessary step. Figure 3 shows the data’s frequency distribution for population mobility reports in five places (pharmacy, parks, transit, workplace, and residential) from 15-Feb-2020 until 14-Jan-2022 with COVID-19-infected cases. From Fig. 3, an example of data will be on day 29 August 2021, the population mobility for pharmacy was 112.778, in parks was 43.40, in transit was 1.54, in the workplace was − 1.4, in residential was -7.93, and the new cases were 255.

**Fig. 4:**
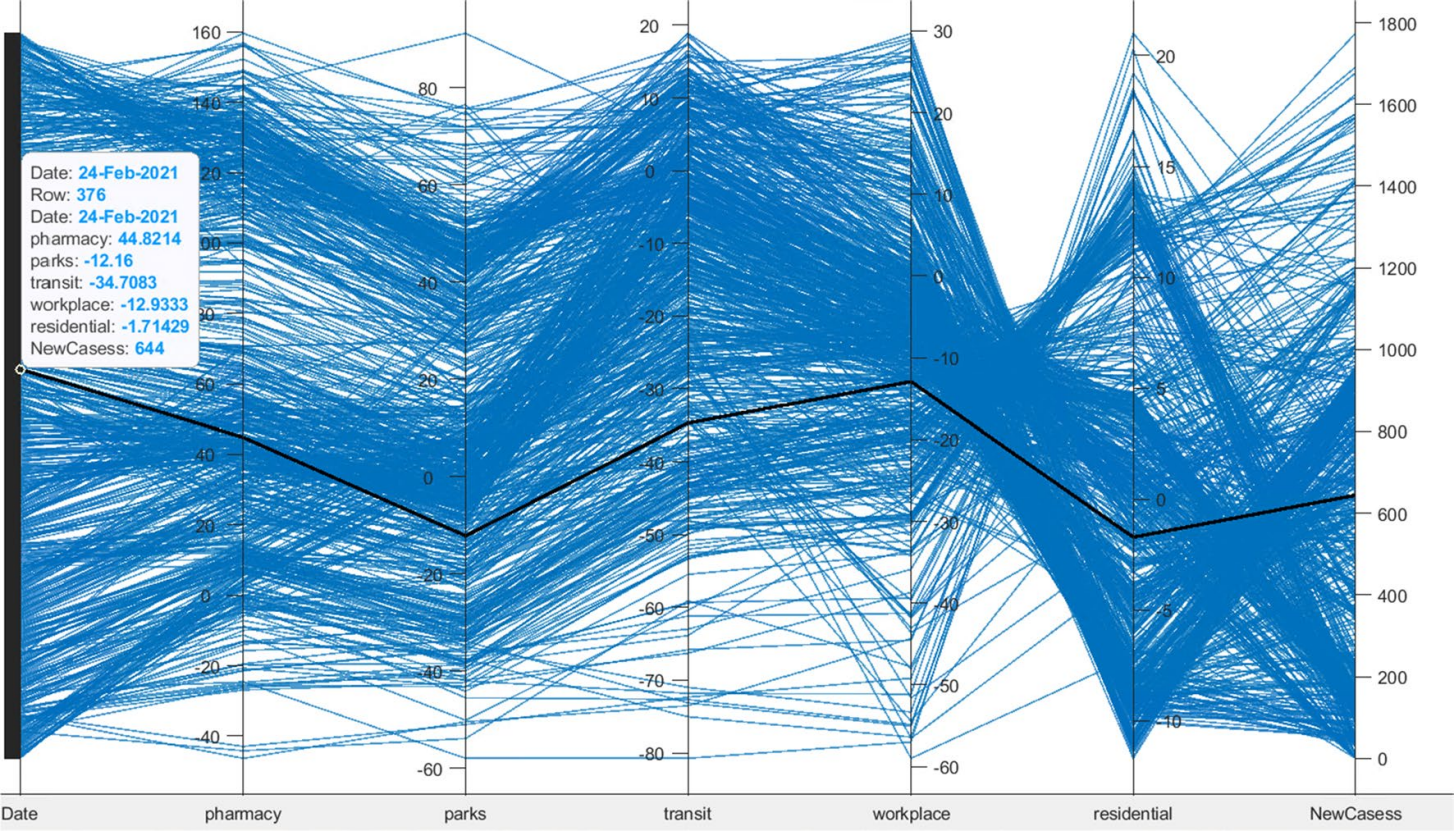
Correlation graph for population mobility reports in five domains (pharmacy, parks, transit, workplace, and residential) from 15-Feb-2020 until 14-Jan-2022 with COVID-19 new cases

Another presentation is the correlation between the two datasets variables which is illustrated in Fig. 4. The figure presents the correlation between population mobility reports on five places (pharmacy, parks, transit, workplace, and residential) and the new COVID-19 new cases from date 15-Feb-2020 until 14-Jan-2022. The bold line presents a sample of data on day 24 February 2021, the population mobility for pharmacy was 44.82, in parks was − 12.16, in transit was − 34.70, in the workplace was − 12.93, in residential was − 1.71, and the new cases were 255.

### Data Preprocessing

The next step is to perform some preliminary processing on the gathered data to be used in the proposed model which will be presented in Sect. 4. Data normalization and standardization were employed during the preprocessing stage [29]. The mean and standard deviation are used to generate values between 0 and 1 after data normalization.

## The Proposed Model Phases and Design

**Fig. 5:**
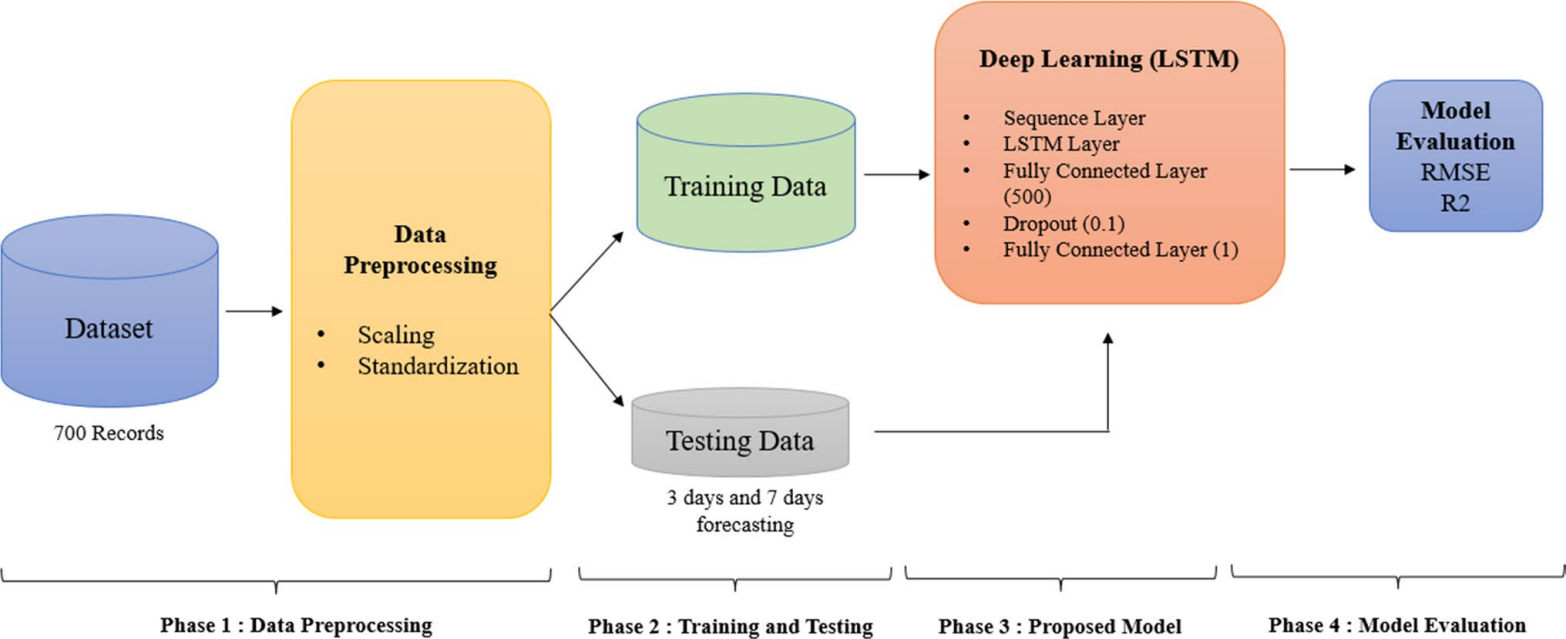
A representation flow of the proposed model design

The proposed model phases block diagram is presented in Fig. 5. The proposed model follows a four-phase process to accomplish this main goal: (1) data preprocessing, (2) data training and testing, (3) deep learning model, and (5) model evaluation measurement.

The data preprocessing was illustrated in Sect. 3.2. The combined dataset was split once for 697 records for the training and 3 records for testing. The second split was for 693 records for training and 7 records for testing. The testing records will be used for measuring the performance of the proposed model. The third phase is the proposed deep learning model which will be presented in the following Sect. 4.1 along with model evaluation in Sect. 4.2.

### The proposed model for deep learning has Proposed Model Design

**Fig. 6:**
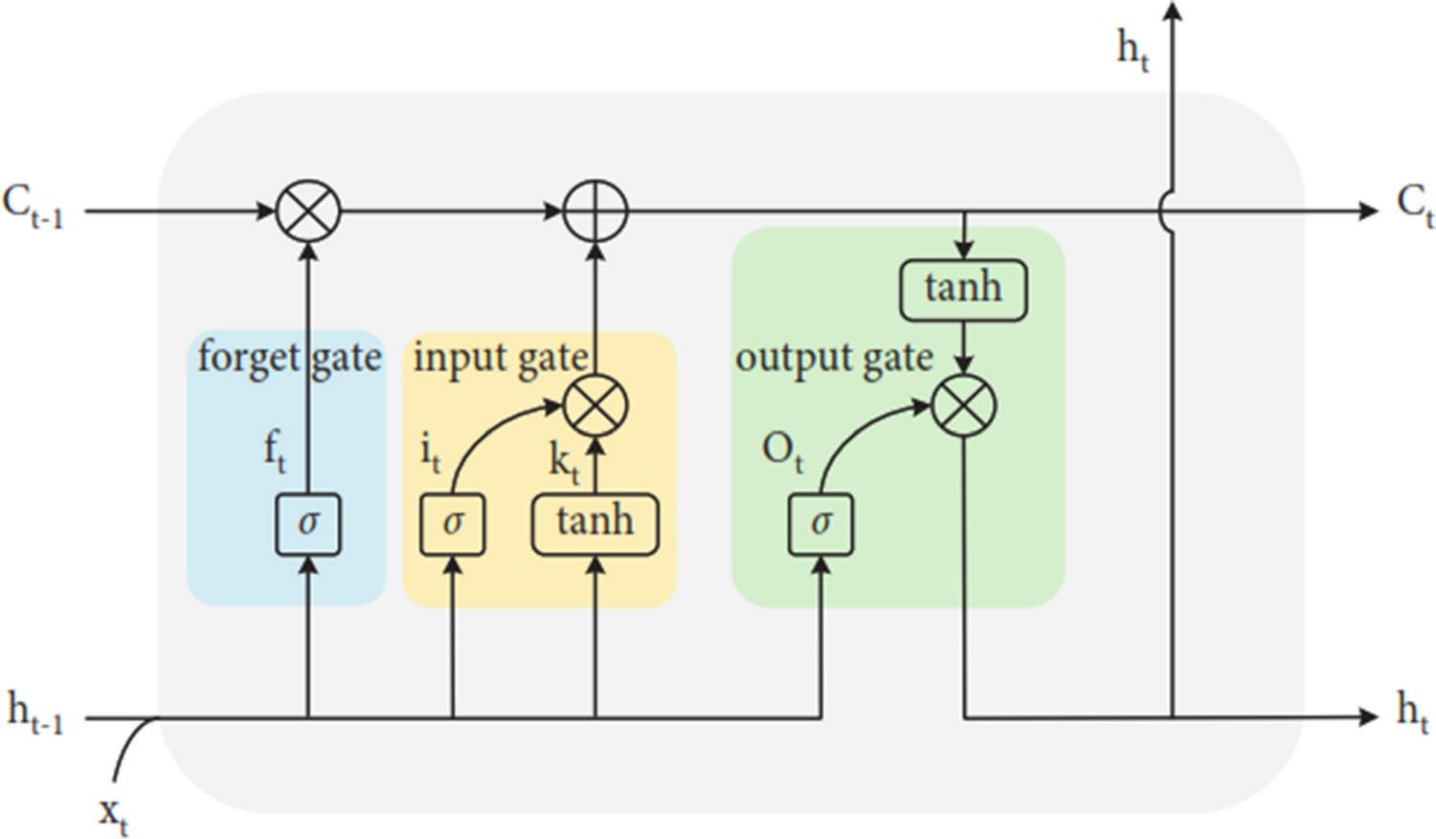
The internal structure of the LSTM network unit [31]

constructed its base design on the long short-term memory LSTM network [30]. Generally, the primary benefit of LSTM over conventional network models is its response connections, which allow data signals to travel backward and improve prediction performance. The LSTM can learn variation from training examples, which is an additional benefit over traditional prediction models. It is suggested to discover the effects of safeguards like contact restriction or shutdown on epidemic incidence by utilizing LSTM’s ability to learn interconnections and remember large amounts of information for long periods [30]. The internal structure of the LSTM network unit is presented in Fig. 6.

The equation of forget gate [31] is presented in Eq. (1):

1 $$f_{t} = \sigma \left( {w_{f} *\left[ {h_{t - 1} ,x_{t} } \right] + b_{f} } \right),$$

where ${f}_{t}$ is the output value of the forget gate, ${h}_{t-1}$ is the output value of the last moment, ${x}_{t}$ is the input value of the current moment, and ${w}_{f}$ and ${b}_{f}$ are the weight matrix and bias vector in the Sigmoid function of the forget gate, respectively. $\left[{h}_{t-1},{x}_{t}\right]$ is the connection matrix of ${h}_{t-1}$ and ${x}_{t}$. The equations of the input gate [31] are presented in Eqs. (2) and (3):

2 $$\begin{gathered} i_{t} = \sigma \left( {w_{i} {*}\left[ {h_{t - 1} ,x_{t} } \right] + b_{i} } \right) \hfill \\ k_{t} = \tan h\left( {w_{k} {*}\left[ {h_{t - 1} ,x_{t} } \right] + b_{k} } \right), \hfill \\ \end{gathered}$$

where $i_{t}$ and $k_{t}$ are the input gate’s output values, and $w_{i}$ and $b_{i}$ are the weight matrix and bias vector in the Sigmoid function of the input gate, respectively; $w_{k}$ and $b_{k}$ are the weight matrix and bias vector in the tanh function of the input gate, respectively.

The equations of the output gate [31] are presented in Eqs. (4) and (5):

3 $$\begin{gathered} O_{t} = \sigma \left( {w_{O} {*}\left[ {h_{t - 1} ,x_{t} } \right] + b_{O} } \right), \hfill \\ h_{t} = O_{t} {\text{*tan}}h\left( {C_{t} } \right), \hfill \\ \end{gathered}$$

where ${O}_{t}$ is the output value of the output gate, ${w}_{O}$ and ${b}_{O}$ are the weight matrix and bias vector in the Sigmoid function of the output gate, respectively, and ${h}_{t}$ is the output value of the current moment. The updated cell state [31] is presented in Eq. (6):

6 $$C_{t} = f_{t} {*}C_{t - 1} + i_{t} {*}k_{t} ,$$

where ${C}_{t}$ is the cell state of the current moment and ${C}_{t-1}$ is the cell state of the last moment.

**Fig. 7:**
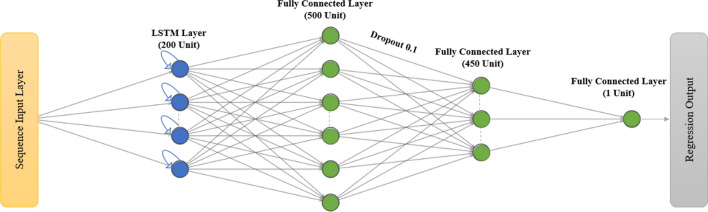
Five layers proposed model design for COVID-19 infection prediction

The proposed model design is presented in Fig. 7. The first layer is the sequence layer, which exposes a temporal dimension to the input data. With a time step of one day, the sequence layer enables the model to identify dependencies between consecutive elements in the sequence and to comprehend recurrent patterns over time.

The second layer is a unidirectional long short-term memory (LSTM) layer, which was integrated to mitigate the problem of vanishing gradients and to foster the model’s ability to maintain and retrieve information across a prolonged period. This enhancement assisted the proposed model in effectively recognizing patterns and dependencies in the input data.

The third layer comprises a fully connected layer of 500 units. It utilizes the outputs from the LSTM layer as inputs to predict the target values. The weights and biases of this layer are learned through the training process, thus enabling the network to make predictions based on the patterns observed in the input data.

The fourth layer is a dropout layer with a rate of 0.1, which serves to avoid overfitting. It randomly drops out 10% of the units in the fully connected layer during each iteration of the training process. This technique helps ensure that the model generalizes well to unseen data rather than memorizing the training data. The final layer is the regression layer. The architecture was developed through multiple trials and by adhering to best practices established in the relevant literature [32].

## Results, Discussion and Performance Analysis

In this section, the experiment environment will be presented along with the results of the experiments. The first subsection will be dedicated to the experiment environment. The second section will be for forecasting results for 3 days, and the third subsection shows results of forecasting for 7 days.

### Experiment Environment

The experiment environment was set up using a Core i5 PC with 32 GB of RAM and a MATLAB 2021b. The following hyperparameters were selected during the experiment according to [32] based on the achieved results which reflected the lowest error possible according to *R*^2^ and RMSE.
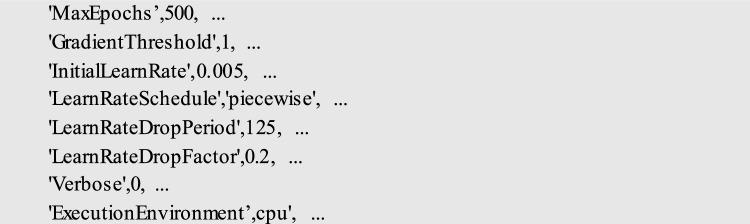


**Fig. 8:**
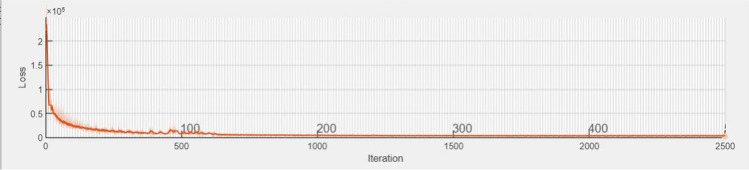
Training loss rate progress during one of the experimental trials

All the experiments are CPU specific with an initial learning rate of 0.005 and the number of training epochs is 500. One of the measures to monitor the performance of the training is the training loss rate. Training loss denotes the discrepancy or deviation between the forecasted output and the factual output of the model throughout the training phase. The progression of the training loss rate implies how the training loss fluctuates over time during the training phase, as the model modifies its parameters to diminish the loss. Typically, the loss rate advancement is demonstrated as a graph or a figure, with the training loss on the *y*-axis, and the training iterations or epochs on the *x*-axis. Figure 8 presents the loss rate progress during one of the training trials.

### Experiment Evaluation Criteria

Both determination coefficient *R*^2^ and RMSE [32] are two statistical indicators to measure the performance of the proposed model. They are used to evaluate the forecasting models’ efficiency and quality of the dataset, which can be determined using those metrics, and each is represented by Eqs. (7) or (8):

7 $$R^{2} = \frac{{\left( {\mathop \sum \nolimits_{i = 1}^{n} \left( {\left( {x_{i} - \overline{x}} \right)\left( {y_{i} - \overline{y}} \right)} \right)} \right)^{2} }}{{\mathop \sum \nolimits_{i = 1}^{n} \left( {x_{i} - \overline{x}} \right)^{2} x \mathop \sum \nolimits_{i = 1}^{n} \left( {y_{i} - \overline{y}} \right)^{2} }},$$

8 $${\text{RMSE}} = \sqrt {\frac{1}{n} \mathop \sum \limits_{i = 1}^{n} \left( {x_{i} - y_{i} } \right)^{2} } ,$$

$n,x,y, \overline{x}\,{\text{and}}\,\overline{y}$ represent the instances number, incidents reported, incidents predicted, average incidents confirmed, and average incidents expected, accordingly.

### Experimental Results for Forecasting 3 Days

**Fig. 9:**
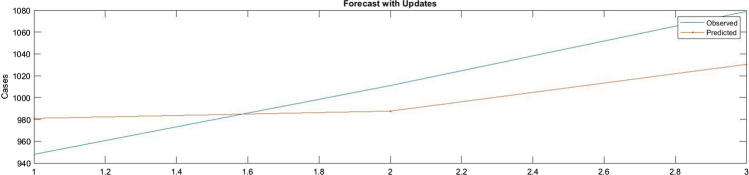
The predicted values of new cases against the actual values for 3 days

**Fig. 10:**
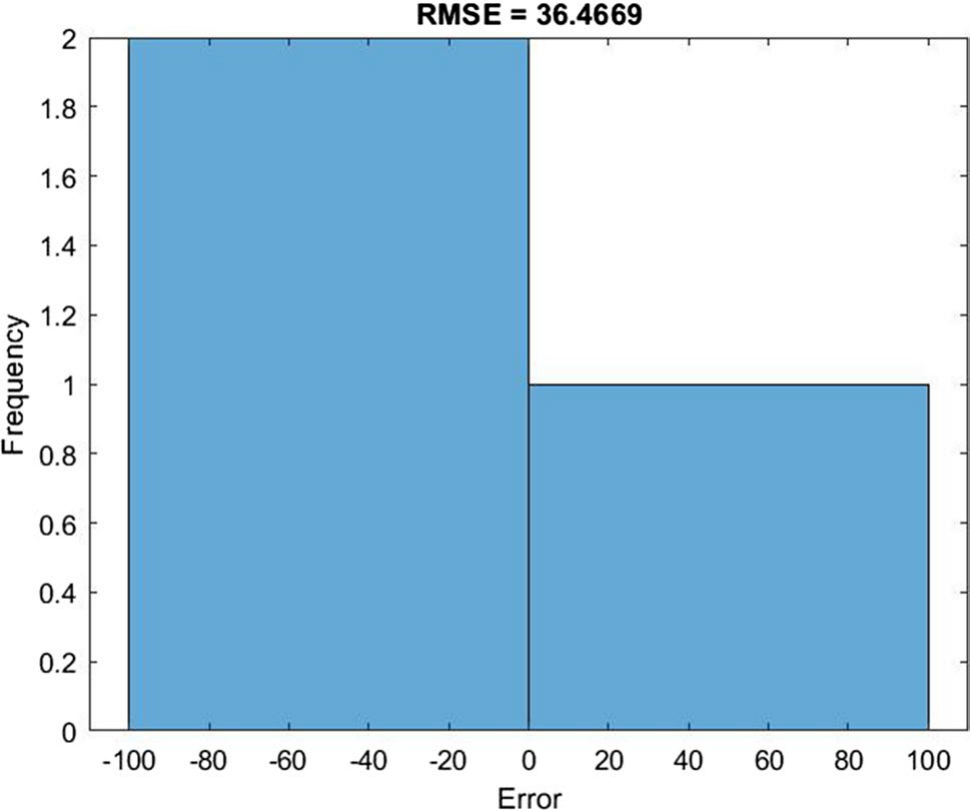
The RMSE graph for 3 days of prediction

Figure 9 presents the difference between the predicted values and the actual values of COVID-19 infection for 3 days. The blue line presents actual values while the red value presents the predicted values. Figure 10 illustrates the RMSE graph for a 3-day prediction. The RMSE was 36.4669, and the *R*^2^ for a 3-day forecast was 96.69%.

### Experimental Results for Forecasting 7 Days

**Fig. 11:**
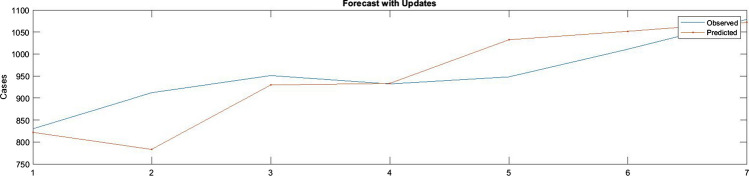
The predicted values of new cases against the actual values for 7 days

**Fig. 12:**
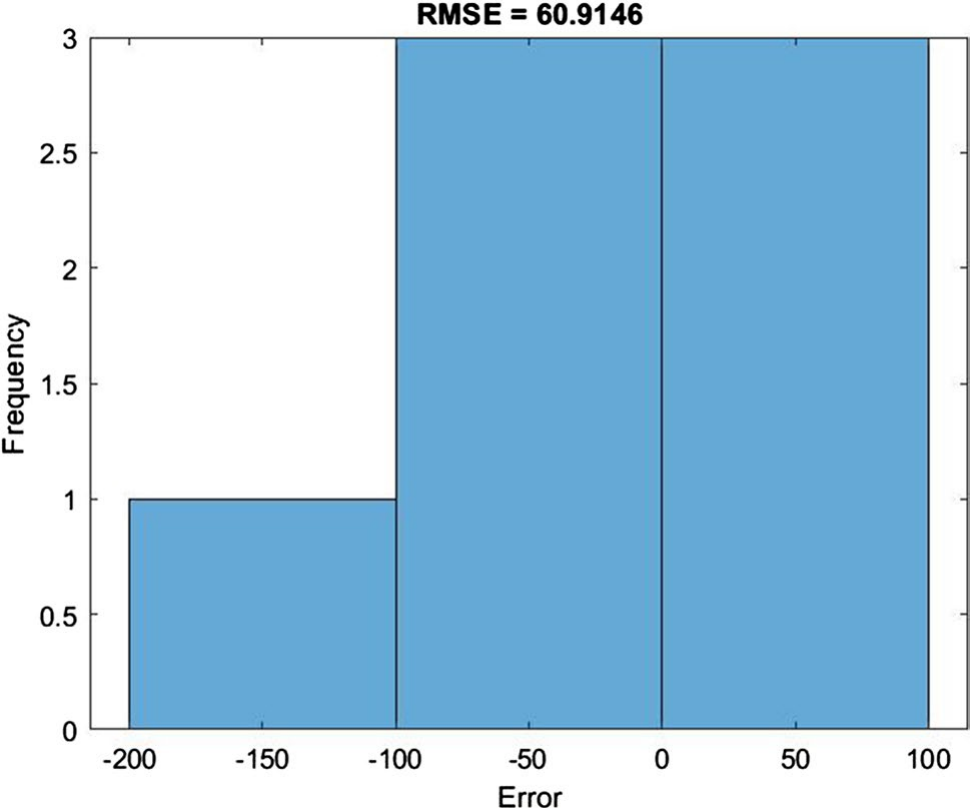
The RMSE graph for 7 days of prediction

Figure 11 presents the difference between the predicted values and the actual values of COVID-19 infection for 7 days. The blue line presents actual values while the red value presents the predicted values. Figure 12 illustrates the RMSE graph for 7 days of prediction. The RMSE was 60.9146. The *R*^2^ for 7 days prediction was 72.71%.

The above results show that the proposed model for the prediction of new infection of COVID-19 cases achieved the highest accuracy possible for prediction for either 3 or 7 days. The highest accuracy was achieved by the model for 3 days of prediction with RMSE 36.46 and *R*^2^ 96.69%. The model still has room for improvement for 7 days of prediction using more data for the training as it achieved RMSE 60.91 and *R*^2^ 72.71%.

## Conclusion and Future Works

COVID-19 has been designated a worldwide health outbreak by the World Health Organization in March 2020. This study presented a COVID-19 infection prediction model in Egypt based on deep learning using population mobility reports. The proposed model performance was tested through the determination of *R*^2^ and RMSE. The following is a summary of the research’s main findings: (a) a new combined dataset from two different sources (Google population mobility reports and newly infected cases by date) was presented, (b) predicting the number of Egyptians who would be infected with the highest accuracy possible 96.69% for 3 days of prediction, (c) predicting the number of Egyptians who would be infected with the highest accuracy possible for 7 days prediction. This study may be able to assist researchers, public health professionals, and politicians in their efforts to anticipate and monitor the Egyptian COVID-19 epidemic depending on mobility reports which are available to be downloaded daily. One of the possible future works is trying other models such as transformers and it could be beneficial to extend the prediction horizon beyond 7 days and compare the accuracy of the model with other existing COVID-19 prediction models. In addition, implementing concept drift across multiple data streams will be an added value to the model to detect the change of error rate.

## Data Availability

The data that support the findings of this study are available from the corresponding author Nour Eldeen Khalifa, upon reasonable request.
